# Supplementary figures and images for: Integrative proteomics and bioinformatic prediction enable a high-confidence apicoplast proteome in malaria parasites
Source: PLoS Biol. 2018 Sep 13;16(9):e2005895. doi: 10.1371/journal.pbio.2005895 (PMC6155542; doi:10.1371/journal.pbio.2005895)

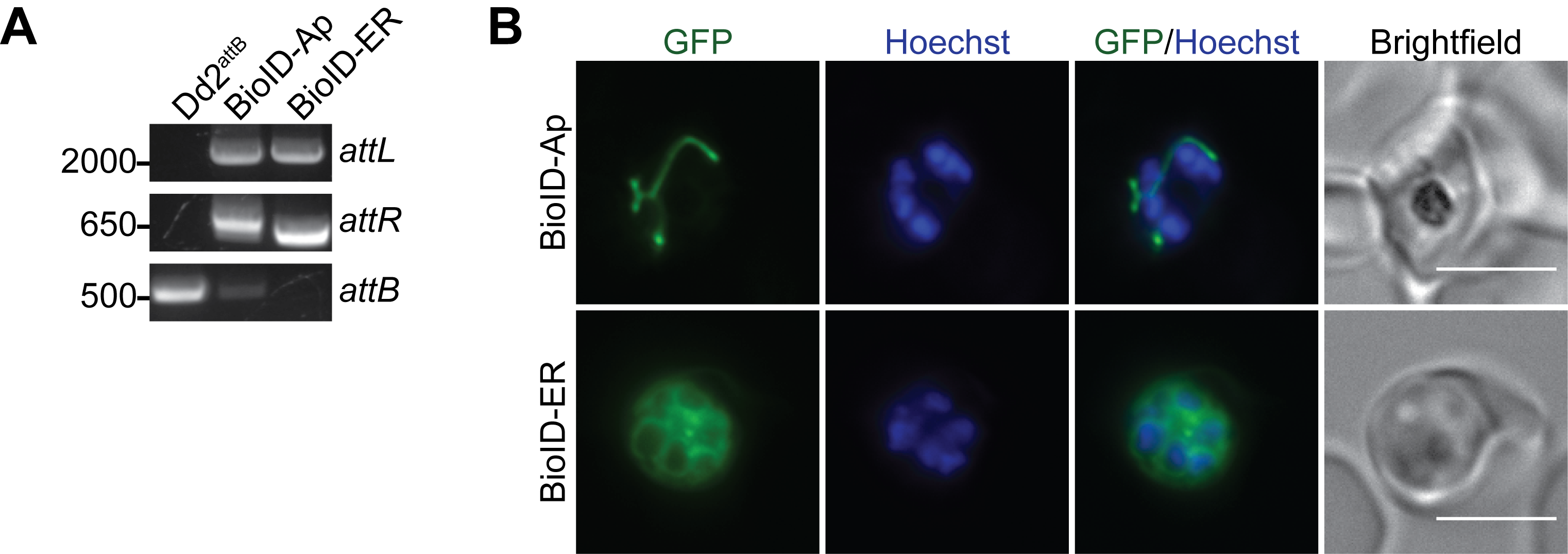

Supplement: S1 Fig — (A) PCR products showing integrated attL and attR sites or unintegrated attB site. (B) Live-cell imaging of Hoechst-stained BioID-Ap and BioID-ER parasites. Scale bars, 5 μm. BioID, proximity-dependent biotin identification; ER, endoplasmic reticulum. (TIF) [file pbio.2005895.s001.tif]

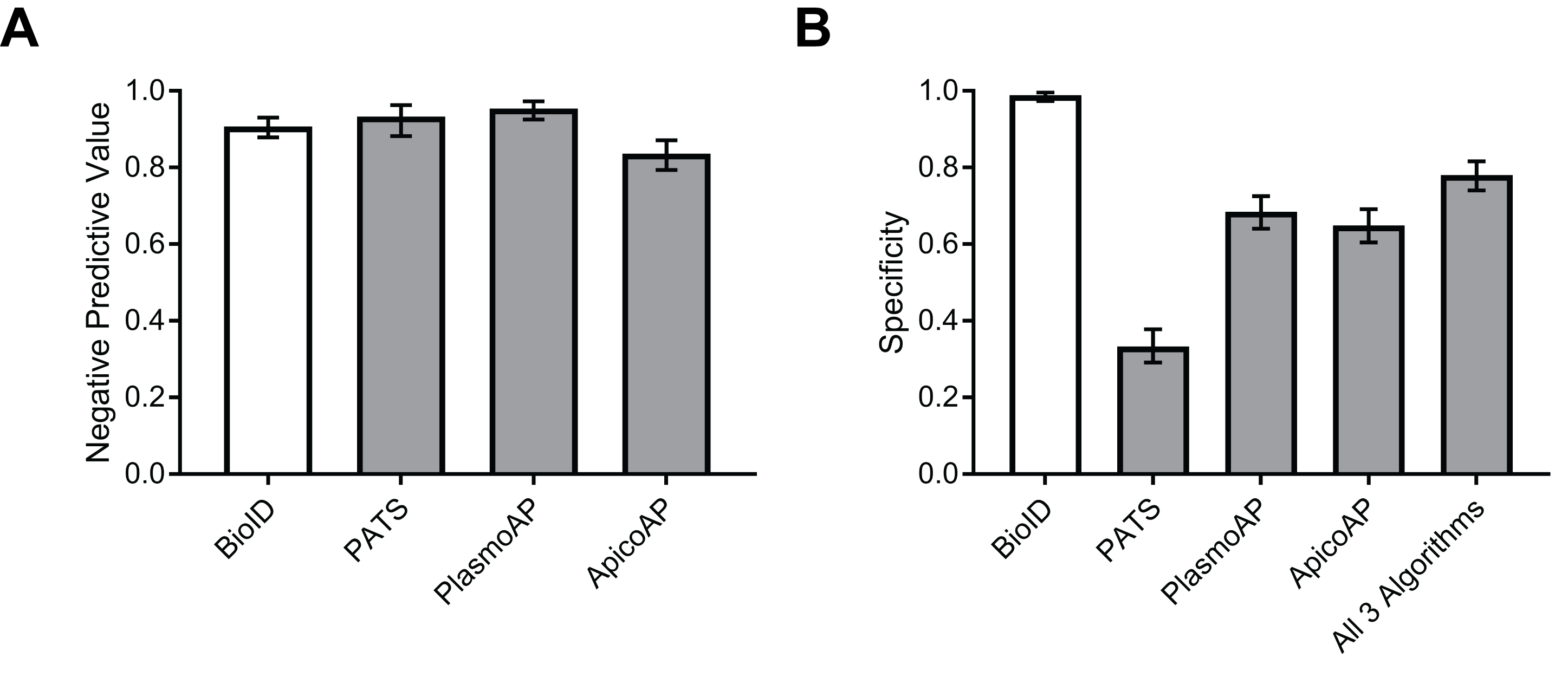

Supplement: S2 Fig — (A) NPV and (B) specificity of apicoplast BioID, PATS, PlasmoAP, and ApicoAP. Error bars represent 95% confidence intervals. Tabulated data are available in S1 Data. ApicoAP, Apicomplexan Apicoplast Proteins algorithm; BioID, proximity-dependent biotin identification; NPV, negative predictive value; PATS, Predict Apicoplast-Targeted Sequences algorithm; PlasmoAP, Plasmodium falciparum Apicoplast-targeted Proteins algorithm. (TIF) [file pbio.2005895.s002.tif]

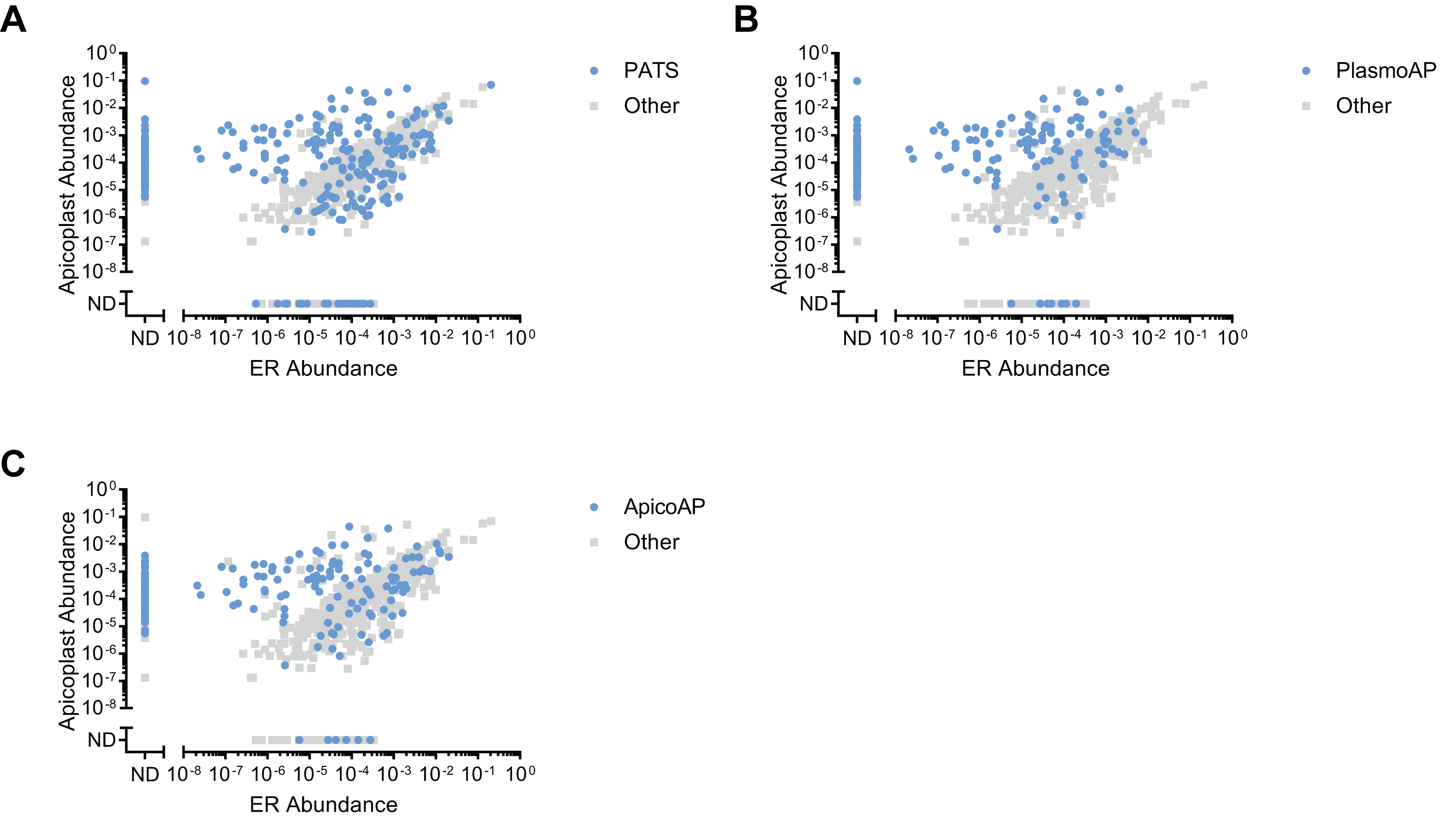

Supplement: S3 Fig — Proteins predicted to localize to the apicoplast by (A) PATS, (B) PlasmoAP, or (C) ApicoAP are highlighted in each graph. Data points are identical to those in Fig 2A. ER, endoplasmic reticulum; ApicoAP, Apicomplexan Apicoplast Proteins algorithm; PATS, Predict Apicoplast-Targeted Sequences algorithm; PlasmoAP, Plasmodium falciparum Apicoplast-targeted Proteins algorithm. (TIF) [file pbio.2005895.s003.tif]

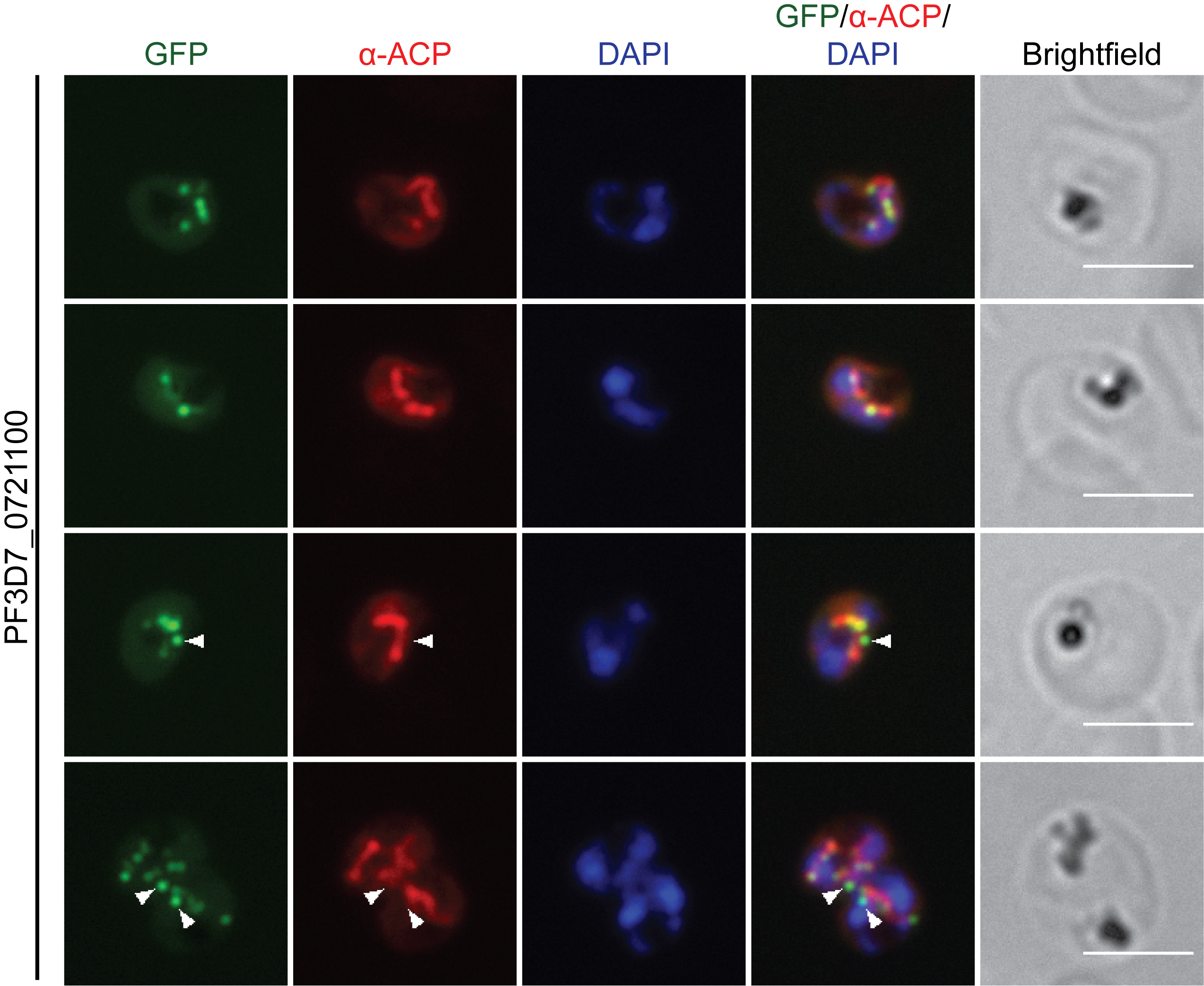

Supplement: S4 Fig — PF3D7_0721100-GFP parasites were stained with an antibody against the apicoplast marker ACP. Arrowheads indicate regions where PF3D7_0721100-GFP puncta appear adjacent to as opposed to colocalizing with ACP. Scale bars, 5 μm. ACP, acyl carrier protein; GFP, green fluorescent protein. (TIF) [file pbio.2005895.s004.tif]

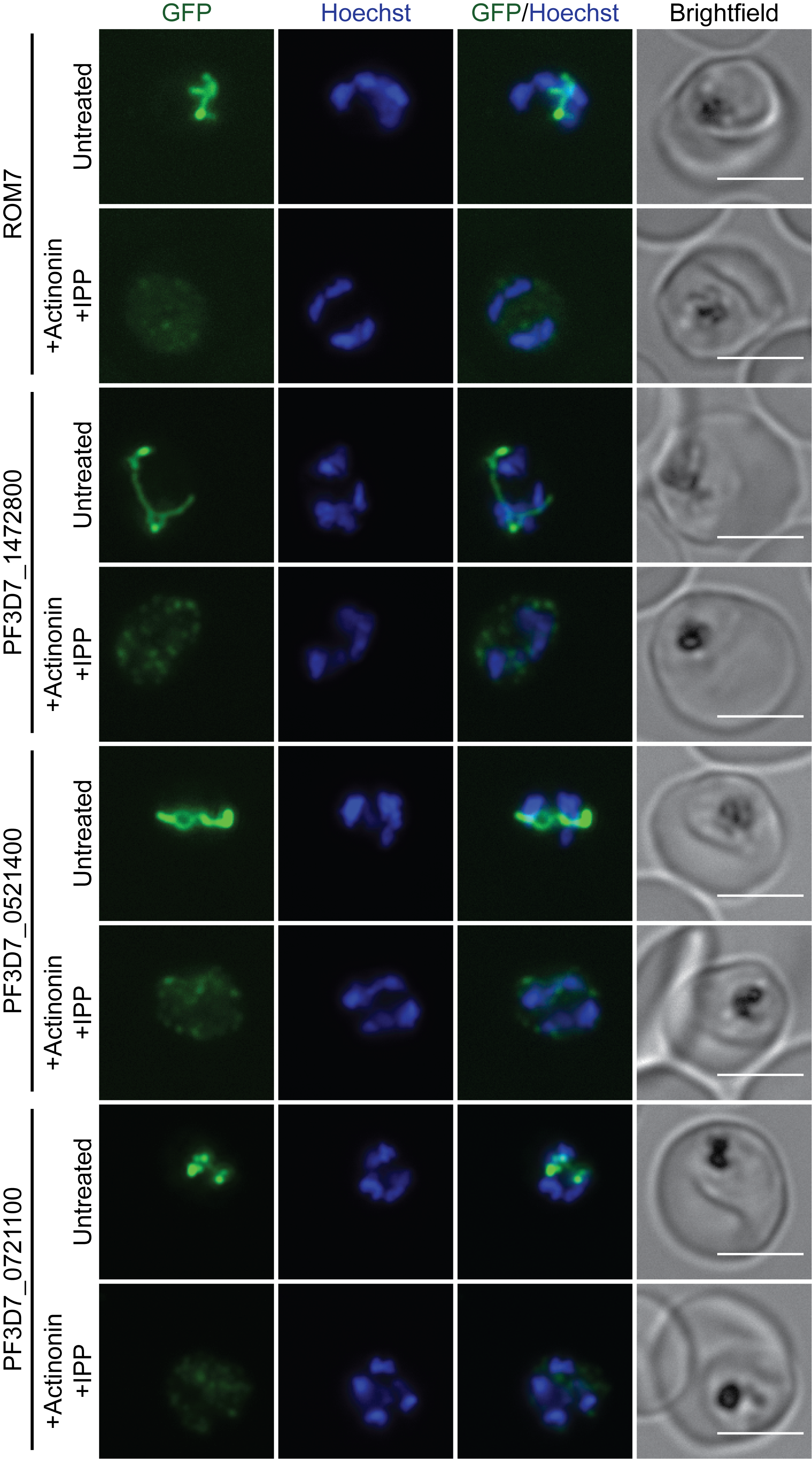

Supplement: S5 Fig — Parasites expressing C-terminally GFP-tagged candidate proteins from apicoplast BioID were either untreated (apicoplast-intact) or treated with 10 μM actinonin/200 μM IPP (apicoplast-disrupted) for 3 days prior to imaging. Scale bars, 5 μm. BioID, proximity-dependent biotin identification; GFP, green fluorescent protein; IPP, isopentenyl pyrophosphate. (TIF) [file pbio.2005895.s005.tif]

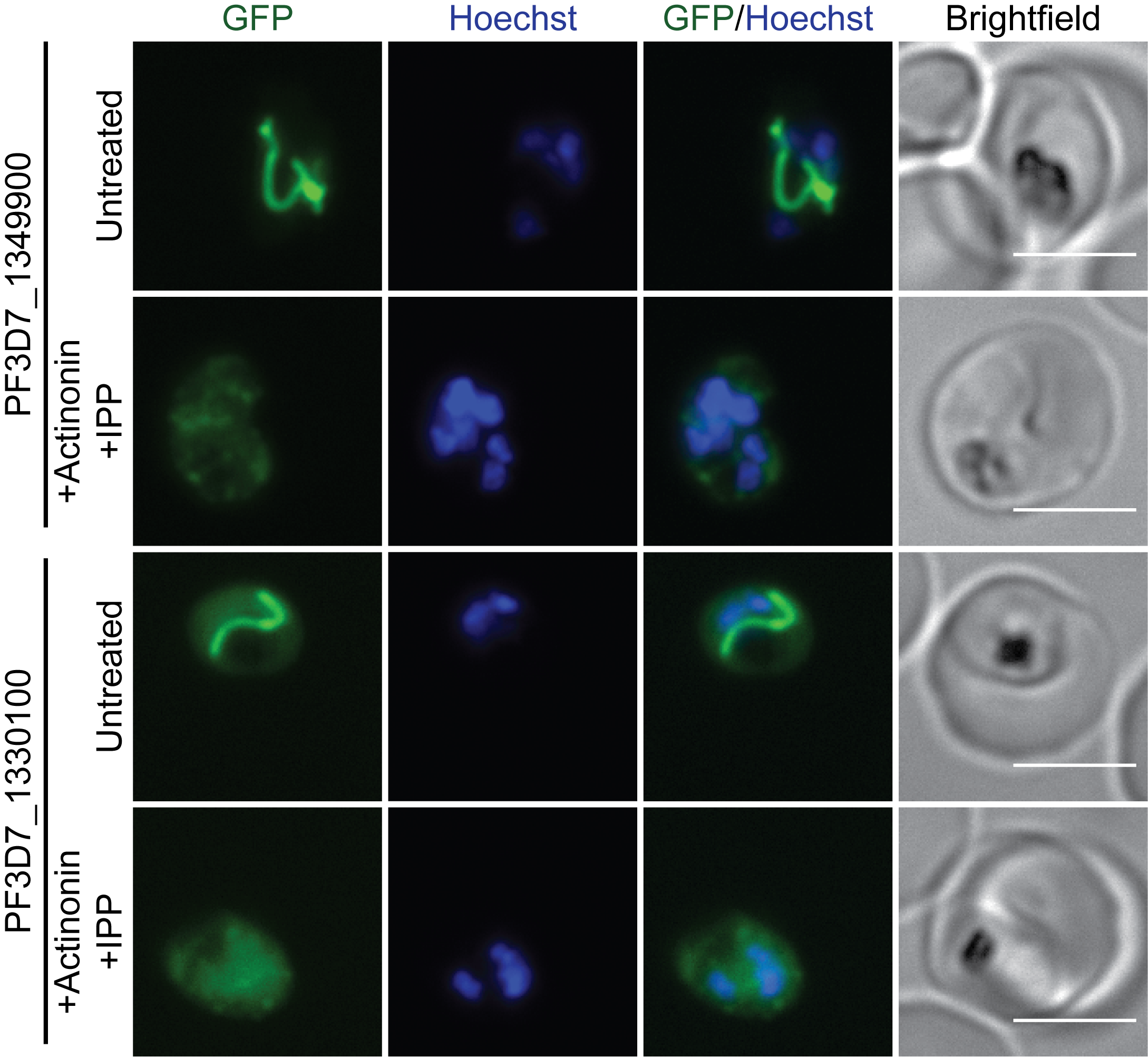

Supplement: S6 Fig — Parasites expressing C-terminally GFP-tagged candidate proteins from PlastNN were either untreated (apicoplast-intact) or treated with 10 μM actinonin/200 μM IPP (apicoplast-disrupted) for 3 days prior to imaging. Scale bars, 5 μm. GFP, green fluorescent protein; IPP, isopentenyl pyrophosphate; PlastNN, Apicoplast Neural Network. (TIF) [file pbio.2005895.s006.tif]

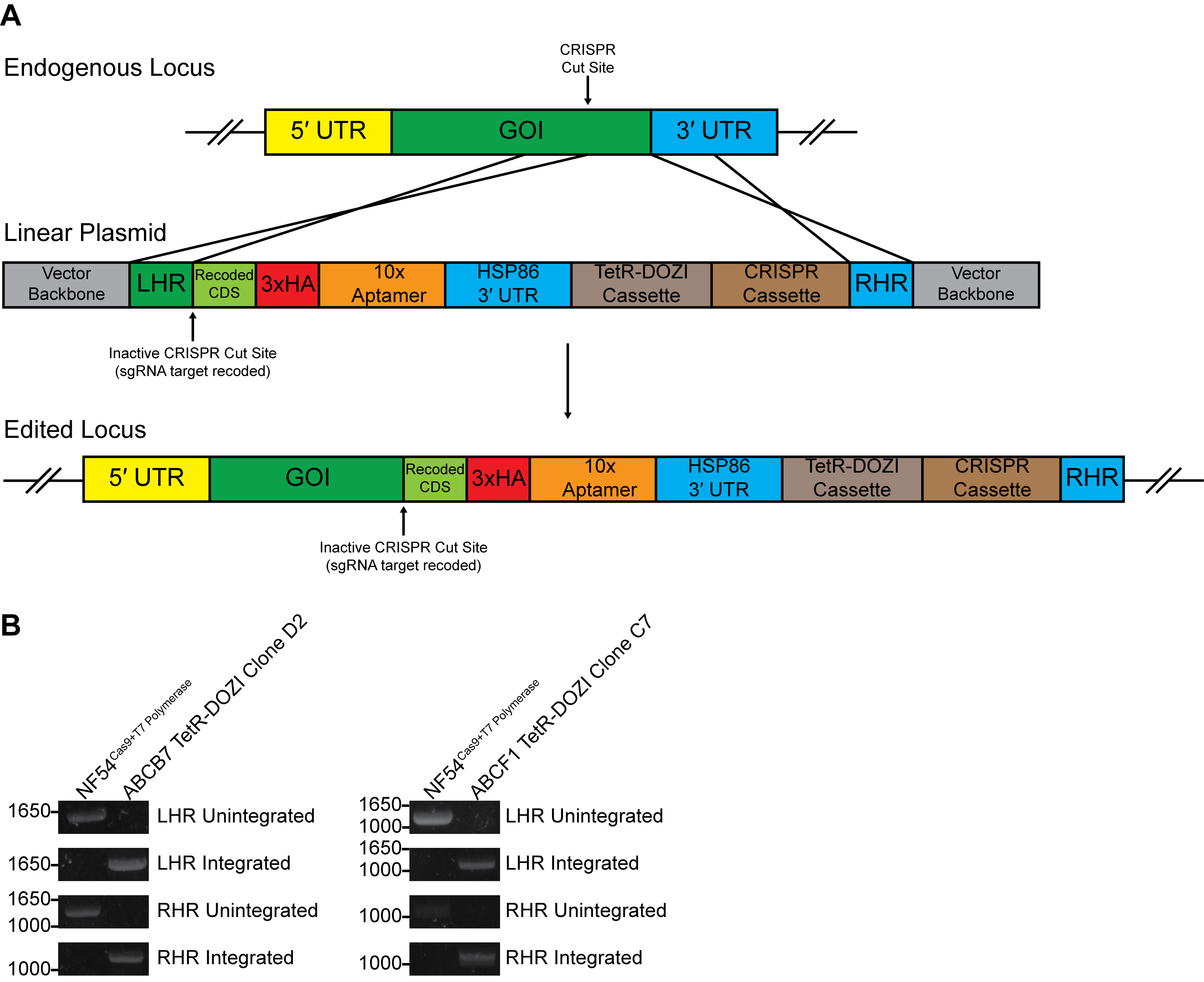

Supplement: S7 Fig — (A) Schematic of CRISPR-Cas9–based endogenous editing to generate conditional knockdown cell lines. (B) PCR products showing integrated or unintegrated LHR and RHR sites in parental NF54Cas9+T7 Polymerase or clonal genome-edited parasites. CRISPR, clustered regularly interspaced short palindromic repeats; DOZI, development of zygote inhibited; GOI, gene of interest; LHR, left homology region; RHR, right homology region; TetR, tetracycline repressor. (TIF) [file pbio.2005895.s007.tif]

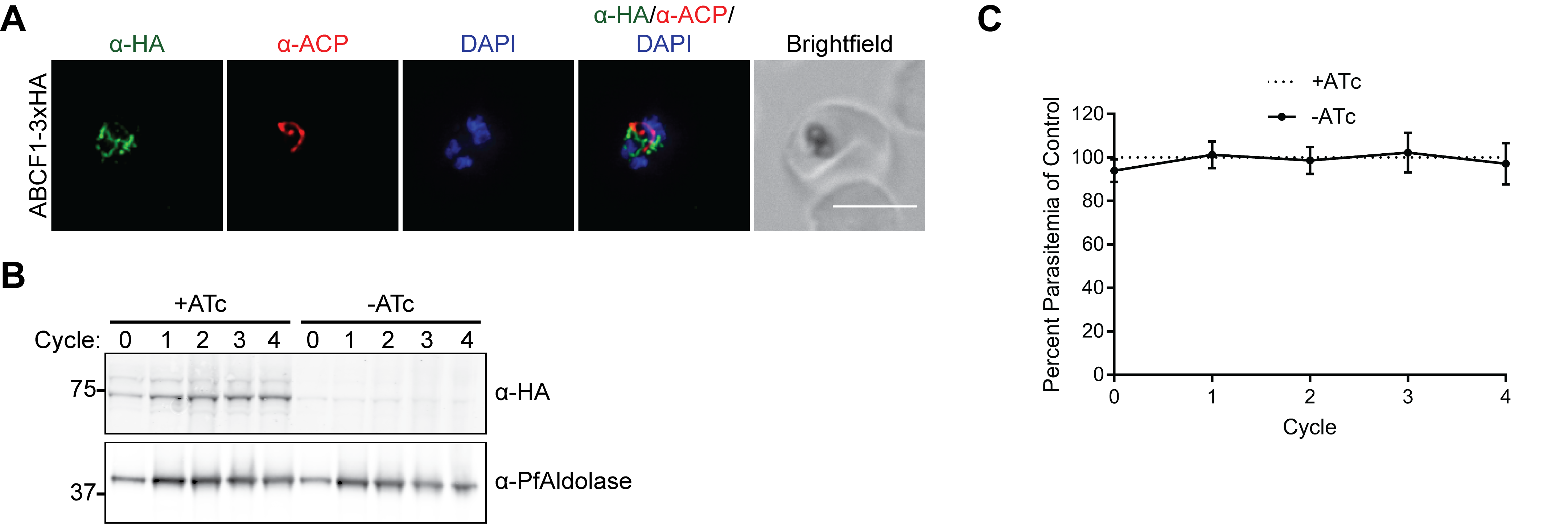

Supplement: S8 Fig — (A) Fixed-cell imaging of ABCB7-3xHA knockdown parasites stained with antibodies raised against the HA tag and the apicoplast marker ACP. Scale bar, 5 μm. (B–C) ABCB7-3xHA knockdown parasites were grown in the presence of ATc (+ATc) or the absence of ATc (−ATc) for 4 growth cycles. (B) Western blot of ABCB7-3xHA expression. (C) Parasite growth. At each time point, data are normalized to the untreated (+ATc) control. Error bars represent standard deviation of the mean of 2 biological replicates. Tabulated data for (C) are available in S1 Data. ABC, ATP-binding cassette; ACP; acyl carrier protein; ATc, anhydrotetracycline; HA, hemagglutinin. (TIF) [file pbio.2005895.s008.tif]
