# Supplementary material for: Integrative proteomics and bioinformatic prediction enable a high-confidence apicoplast proteome in malaria parasites
Source: PLoS Biol. 2018 Sep 13;16(9):e2005895. doi: 10.1371/journal.pbio.2005895 (PMC6155542; doi:10.1371/journal.pbio.2005895)
Supplement: S5 Table — (DOCX) [file pbio.2005895.s013.docx]

**S5 Table.** Layer dimensions for PlastNN neural network.

| **Layer** | Input | Hidden 1 | Hidden 2 | Hidden 3 | Output |
| --- | --- | --- | --- | --- | --- |
| **Dimension** | 28 | 64 | 64 | 16 | 2 |
| **Non-linearity** | - | ReLU | ReLU | ReLU | Softmax |
