# Supplementary material for: Integrative proteomics and bioinformatic prediction enable a high-confidence apicoplast proteome in malaria parasites
Source: PLoS Biol. 2018 Sep 13;16(9):e2005895. doi: 10.1371/journal.pbio.2005895 (PMC6155542; doi:10.1371/journal.pbio.2005895)
Supplement: S6 Table — (DOCX) [file pbio.2005895.s014.docx]

**S6 Table.** Performance of different models in cross-validation.

| **Model** | **Features** | **Hyper- parameters** | **Accuracy** | | **Sensitivity** | | **Specificity** | | **NPV** | | **PPV** | |
| --- | --- | --- | --- | --- | --- | --- | --- | --- | --- | --- | --- | --- |
|  |  |  | **Mean** | **SD** | **Mean** | **SD** | **Mean** | **SD** | **Mean** | **SD** | **Mean** | **SD** |
| Logistic regression | Amino acid frequencies |  | 0.89 | 0.04 | 0.82 | 0.05 | 0.92 | 0.04 | 0.92 | 0.02 | 0.84 | 0.08 |
|  | Transcriptome |  | 0.81 | 0.02 | 0.51 | 0.10 | 0.95 | 0.04 | 0.81 | 0.03 | 0.84 | 0.11 |
|  | Amino acid frequencies + transcriptome | LASSO penalty; lambda = 0.005 | 0.91 | 0.03 | 0.85 | 0.06 | 0.93 | 0.04 | 0.94 | 0.04 | 0.87 | 0.05 |
| Neural network | Amino acid frequencies | L2 regularization; 0.05  Learning rate = 0.001 | 0.91 | 0.02 | 0.83 | 0.09 | 0.95 | 0.03 | 0.92 | 0.04 | 0.87 | 0.08 |
|  | Transcriptome |  | 0.90 | 0.02 | 0.91 | 0.05 | 0.90 | 0.04 | 0.96 | 0.02 | 0.80 | 0.06 |
|  | Amino acid frequencies + transcriptome |  | 0.96 | 0.02 | 0.95 | 0.05 | 0.98 | 0.02 | 0.97 | 0.03 | 0.95 | 0.04 |
