# Supplementary material for: Integrative proteomics and bioinformatic prediction enable a high-confidence apicoplast proteome in malaria parasites
Source: PLoS Biol. 2018 Sep 13;16(9):e2005895. doi: 10.1371/journal.pbio.2005895 (PMC6155542; doi:10.1371/journal.pbio.2005895)
Supplement: S9 Table — (DOCX) [file pbio.2005895.s017.docx]

**S9 Table.** Summary of BioID and PlastNN candidate localization data from this study and Sayers et al.

| **Gene ID** | **Gene Name or Annotation** | **Identified by BioID or PlastNN?** | **Source of Localization Data** | **Localization Result** |
| --- | --- | --- | --- | --- |
| PF3D7_1358300 | ROM7 | BioID | This study | Apicoplast |
| PF3D7_1472800 | Conserved *Plasmodium* protein, unknown function | BioID | This study | Apicoplast |
| PF3D7_0521400 | Conserved *Plasmodium* protein, unknown function | BioID | This study | Apicoplast |
| PF3D7_0721100 | Conserved *Plasmodium* protein, unknown function | BioID | This study | Inconclusive |
| PF3D7_1209900 | ABCB7 | BioID | This study | Most likely non-apicoplast |
| PF3D7_0813700 | ABCF1 | BioID | This study | Apicoplast |
| PF3D7_1145500 | ABCB3 | BioID | Sayers et al. | Apicoplast |
| PF3D7_0302600 | ABCB4 | BioID | Sayers et al. | Apicoplast |
| PF3D7_1021300 | Apicoplast integral membrane protein, putative | BioID | Sayers et al. | Apicoplast |
| PF3D7_1349900 | Conserved *Plasmodium* protein, unknown function | PlastNN | This study | Apicoplast |
| PF3D7_1330100 | Conserved *Plasmodium* protein, unknown function | PlastNN | This study | Apicoplast |
| PF3D7_0908100 | Apicoplast integral membrane protein, putative | PlastNN | Sayers et al. | Apicoplast |
